# Genomic insights into the beneficial potential of Bifidobacterium and Enterococcus strains isolated from Cameroonian infants

**DOI:** 10.1099/mgen.0.001354

**Published:** 2025-02-19

**Authors:** Pierre Marie Kaktcham, Magdalena Kujawska, Edith Marius Foko Kouam, Laverdure Tchamani Piame, Michele Letitia Tchabou Tientcheu, Julia Mueller, Angela Felsl, Bastian-Alexander Truppel, François Zambou Ngoufack, Lindsay J. Hall

**Affiliations:** 1 Research Unit of Biochemistry of Medicinal Plants, Food Science and Nutrition (URBPMAN) – Department of Biochemistry, Faculty of Science, University of Dschang, Cameroon. P.O Box 67, Dschang, Cameroon; 2 Intestinal Microbiome, ZIEL – Institute for Food & Health, Technical University of Munich, Freising, 85354, Germany; 3 Department of Microbes, Infection and Microbiomes, School of Infection, Inflammation and Immunology, College of Medicine and Health, University of Birmingham, Birmingham, B15 42TT, UK; 4 Department of Physiological Sciences and Biochemistry, Faculty of Medicine and Pharmaceutical Sciences, University of Dschang, Dschang, Cameroon; 5 Food, Microbiome & Health, Quadram Institute Bioscience, Norwich Research Park, Norwich, NR4 7UQ, UK; 6 Norwich Medical School, University of East Anglia, Norwich Research Park, NR4 7TJ, Norwich, UK

**Keywords:** bifidobacteria, *Enterococcus*, functional genomics, probiotics, safety, whole-genome sequencing

## Abstract

A healthy early-life gut microbiota plays an important role in maintaining immediate and long-term health. Perturbations, particularly in low- to middle-income communities, are associated with increased infection risk. Thus, a promising avenue for restoring a healthy infant microbiota is to select key beneficial bacterial candidates from underexplored microbiomes for developing new probiotic-based therapies. This study aimed to recover bifidobacteria and lactic acid bacteria from the faeces of healthy Cameroonian infants and unravel the genetic basis of their beneficial properties. Faecal samples were collected from 26 infants aged 0–5 months recruited in Dschang (Cameroon). Recovered bacterial isolates were subjected to whole-genome sequencing and *in silico* analysis to assess their potential for carbohydrate utilization, their antimicrobial capacities, host-adaptation capabilities and their safety. From the range of infant-associated *Bifidobacterium* and *Enterococcus* strains identified, *Bifidobacterium* species were found to harbour putative gene clusters implicated in human milk oligosaccharide metabolism. Genes linked to the production of antimicrobial peptides such as class IV lanthipeptides were found in *Bifidobacterium pseudocatenulatum*, while those implicated in biosynthesis of cytolysins, enterolysins, enterocins and propeptins, among others, were identified in enterococci. Bifidobacterial isolates did not contain genes associated with virulence; however, we detected the presence of putative tetracycline resistance genes in several strains belonging to *Bifidobacterium animalis* subsp. *lactis* and *Bifidobacterium longum* subsp. *longum*. Among the enterococci, *Enterococcus mundtii* PM10 did not carry any genes associated with antimicrobial resistance or virulence. The latter, together with all the *Bifidobacterium* strains, also encoded several putative adaptive and stress-response-related genes, suggesting robust gastroinstestinal tract colonization potential. This work provides the first genomic characterization of *Bifidobacterium* and *Enterococcus* isolates from Cameroonian infants. Several strains showed the genomic potential to confer beneficial properties. Further phenotypic and clinical investigations are needed to confirm their suitability as customized probiotics.

­

**Impact Statement**

The underexplored ecological niches, e.g. the gut microbiota of infants from low- and middle-income countries (LMICs), could represent a reservoir of novel population-specific beneficial microbes. In this study, we recovered bifidobacteria and enterococci from the intestinal microbiota of Cameroonian infants. Their genomic characterization revealed that strains belonging to *Bifidobacterium bifidum*, *Bifidobacterium breve*, *Bifidobacterium longum* subsp. *longum*, *Bifidobacterium pseudocatenulatum*, *Bifidobacterium animalis* subsp. *lactis* and *Enterococcus mundtii* have the potential to be considered for further studies aiming at the development of customized probiotics from Cameroon, in particular, and LMICs in general. Not only does our work constitute the first report of the isolation and genomic characterization of bifidobacteria and enterococci from Cameroonian infants, but it also contributes to the reduction of the scarcity of information available on the bacterial members of the infant intestinal microbiota in LMICs.

## Data Summary

The draft genomes of 36 infant-associated isolates sequenced here have been deposited to the National Center for Biotechnology Information (NCBI) database under the BioProject number PRJNA1091569. Individual accession numbers are available in Table 1. Here is the DOI number of the material stored in figshare: https://doi.org/10.6084/m9.figshare.28278884 [1].

**Table 1.** Putative gene clusters encoding for secondary metabolites in *Bifidobacterium* and *Enterococcus* genomes based on antiSMASH and BAGEL4 analyses

	Strain	Accession no.	Sample ID	Bacteriocin/RiPP identified (antiSMASH)	Bacteriocin/RiPP identified (BAGEL4)
1	*Bifidobacterium animalis* subsp. *lactis*LH-CAM-01	JBDXST000000000	V11	No	No
2	*Bifidobacterium animalis* subsp. *lactis*LH-CAM-04	JBDXSQ000000000	V22	No	No
3	*Bifidobacterium animalis* subsp. *lactis* LH-CAM-58	JBDXSD000000000	V21-2	No	No
4	*Bifidobacterium bifidum* LH-CAM-42	JBDXSJ000000000	V14-5	No	No
5	*Bifidobacterium breve* LH-CAM-38	JBDXSK000000000	V14-1	No	No
6	*Bifidobacterium breve* LH-CAM-52	JBDXSG000000000	V17-5	No	No
7	*Bifidobacterium breve* LH-CAM-63	JBDXSC000000000	V23-2	No	No
8	*Bifidobacterium longum* subsp. *longum* LH-CAM-03	JBDXSR000000000	V20	No	No
9	*Bifidobacterium longum* subsp. *longum* LH-CAM-33	JBDXSL000000000	V13-1	No	No
10	*Bifidobacterium pseudocatenulatum* LH-CAM-02	JBDXSS000000000	V15	Lanthipeptide class IV	No
11	*Enterococcus faecalis* LH-CAM-21	JBDXSP000000000	V8	Lanthipeptide class II (cytolysin ClyLI/cytolysin ClyLs)	Cytolysin ClyLIEnterolysin AEnterocin SE-K4
12	*Enterococcus faecalis* LH-CAM-23	JBDXSO000000000	V7-2	Lanthipeptide class II (cytolysin ClyLI/cytolysin ClyLs)	Cytolysin ClyLIEnterolysin AEnterocin SE-K4
13	*Enterococcus faecalis* LH-CAM-25	JBDXSN000000000	V22	Sactipeptides	No
14	*Enterococcus faecalis* LH-CAM-32	JBDXSM000000000	V12-2	Sactipeptides	Enterolysin AEnterocin SE-K4Propeptin 2
15	*Enterococcus faecalis* LH-CAM-43	JBDXSI000000000	V16	No	No
16	*Enterococcus faecalis* LH-CAM-51	JBDXSH000000000	V17-4	No	Enterolysin A
17	*Enterococcus faecalis* LH-CAM-54	JBDXSF000000000	V18-2	No	Enterolysin A
18	*Enterococcus faecalis* LH-CAM-56	JBDXSE000000000	V19	Lanthipeptide class II (cytolysin ClyLI/cytolysin ClyLs)	Enterocin SE-K4Enterocin 96Cytolysin ClyLIEnterolysin APropeptin 2
19	*Enterococcus faecalis* LH-CAM-67	JBDXSB000000000	V24-1	02 sactipeptides	Enterocin SE-K4Enterocin P
20	*Enterococcus faecalis* LH-CAM-70	JBDXSA000000000	V25-2	Lanthipeptide class II (cytolysin ClyLI/cytolysin ClyLs)Sactipeptides	Enterocin SE-K4Cytolysin ClyLIEnterolysin APropeptin
21	*Enterococcus faecalis* PM1	JBDXRZ000000000	V1	No	Propeptin 2
22	*Enterococcus faecalis* PM11	JBDXRX000000000	V16	No	No
23	*Enterococcus faecalis* PM12	JBDXRW000000000	V17	No	No
24	*Enterococcus faecalis* PM14	JBDXRV000000000	V18	Lanthipeptide class II (cytolysin ClyLI/cytolysin ClyLs)Sactipeptides	Enterocin SE-K4Enterocin 96Cytolysin ClyLIEnterolysin APropeptin 2
25	*Enterococcus faecalis* PM2	JBDXRU000000000	V1	Lanthipeptide class II (cytolysin ClyLI/cytolysin ClyLs)	Enterocin SE-K4Cytolysin ClyLIEnterolysin APropeptin 2
26	*Enterococcus faecalis* PM20	JBDXRT000000000	V21	Lanthipeptide class II (cytolysin ClyLI/cytolysin ClyLs)Sactipeptides	Enterocin SE-K4Enterocin 96Cytolysin ClyLIEnterolysin A
27	*Enterococcus faecalis* PM27	JBDXRQ000000000	V23	Lanthipeptide class II (cytolysin ClyLI/cytolysin ClyLs)Sactipeptides	Enterocin SE-K4Enterocin 96Cytolysin ClyLIEnterolysin A
28	*Enterococcus faecalis* PM3	JBDXRP000000000	V16	Lanthipeptide class II (cytolysin ClyLI/cytolysin ClyLs)	Enterocin SE-K4Cytolysin ClyLIEnterolysin APropeptin 2
29	*Enterococcus faecalis* PM30	JBDXRO000000000	V25	Lanthipeptide class II (cytolysin ClyLI/cytolysin ClyLs)Sactipeptides	Enterocin SE-K4Enterocin 96Cytolysin ClyLIEnterolysin A
30	*Enterococcus faecalis* PM5	JBDXRL000000000	V6	No	No
31	*Enterococcus faecalis* PM6	JBDXRK000000000	V8	Sactipeptides	No
32	*Enterococcus faecium* PM21	JBDXRS000000000	V21	T3PKS	Bac 32SactipeptidesEnterocin AEnterolysin A
33	*Enterococcus faecium* PM25	JBDXRR000000000	V22	T3PKSEnterocin ASactipeptides	Enterocin AUViBEnterolysin A
34	*Enterococcus faecium* PM32	JBDXRN000000000	V26	T3PKS	Bac 32SactipeptidesEnterocin AEnterolysin A
35	*Enterococcus faecium* PM34	JBDXRM000000000	V17	T3PKS	Bac 32SactipeptidesEnterocin AEnterolysin A
36	*Enterococcus mundtii* PM10	JBDXRY000000000	V16	T3PKSTerpene	SactipeptidesUViBEnterolysin A

## Introduction

The gut microbiota plays an essential role in human health, principally in host metabolism, physiology, nutrition and immune function [2,3]. Especially during early life, the microbiota performs crucial roles in infant development that determine their health status in childhood and adulthood [4,6]. For example, members of the early-life microbiota can break down non-fermentable sugars such as human milk oligosaccharides (HMOs) and complex plant carbohydrates to produce short-chain fatty acids (SCFAs) [7,8]. These compounds have been shown to contribute to the shaping of the immune system and the metabolic, endocrine and other host developmental pathways, as well as preventing colonization by pathogenic micro-organisms [9,10].

The very dynamic infant-associated microbiota only begins to stabilize towards the third year of life, and its perturbation has been associated with conditions such as atopy, asthma, diabetes and gastrointestinal conditions such as diarrhoea, inflammatory bowel diseases and necrotizing enterocolitis [11,12]. Within the African context, this imbalance may be caused by several factors including host genetics, maternal diet and maternal microbiota composition, antibiotic exposure and malnutrition and poor hygiene and sanitation practices [13,14]. To mitigate this serious health problem in low- and middle-income countries (LMICs), probiotics, prebiotics and synbiotics are widely used as therapeutic options [15,16].

Probiotics are ‘live micro-organisms that, when administered in adequate amounts, confer a health benefit on the host’ [17]. The most widely studied and used probiotic strains belong to lactic acid bacteria (LAB) of genera *Lactobacillus*, *Lacticaseibacillus*, *Lactiplantibacillus*, *Levilactobacillus*, *Ligilactobacillus*, *Limosilactobacillus* and to the genus *Bifidobacterium*, which have been shown to dominate the early-life microbiota of humans and animals [18,19] and to display health-promoting and disease-preventing traits. Another member of the LAB group and a common gut commensal is *Enterococcus*, which demonstrates both probiotic and potentially pathogenic characteristics [20]. Despite this duality, many *Enterococcus* strains have been shown to be effective and safe and have thus been used as probiotics in humans and animals. Mounting evidence has reported the success of particular strains of LAB and *Bifidobacterium* in modulating the gut microbiota and preventing, treating or reducing the risk of microbiota-associated perturbation-related disorders or diseases [21,22]. Among the mechanisms by which they exert beneficial effects are their abilities to degrade particular dietary components, modulation of gut epithelial activity, increase in mucin production, microbial communication, production of antimicrobial substances (mainly SCFAs and bacteriocins) and immune system modulation [23,26].

Given the pivotal role of bifidobacteria and LAB in improving/maintaining health, coupled with the acute rise in antimicrobial resistance (AMR) among pathogenic bacteria, there is an increasing trend in the probiotic market for new target strains with more efficient probiotic potential, antimicrobial activity [but lack of AMR genes (ARG)] and immunomodulatory anti-infection capacities. In this respect, a promising avenue for novel strain development is an exploration of different geographical regions and ecological niches. One such niche is the early-life microbiota of non-Western communities that remain understudied, from which data and information on microbial composition are scarce. To the best of our knowledge, the microbiomes of Cameroonian infants living in rural areas have not yet been extensively profiled and may constitute a reservoir of key bifidobacteria and LAB candidates.

The search for novel probiotics currently involves the use of *in silico*-based approaches alongside traditional culture-dependent ones as they provide a deeper understanding and insight into the full breadth of strains’ biological capabilities in terms of their probiotic, functional and safety-associated capacities [27,28]. Indeed, the analysis of genomic data helps to understand the potential mechanisms by which bacteria adapt to the specific environment of the gastrointestinal tract (GIT), while also revealing genetic functions that mediate specific microbe-microbe and microbe-host interactions – features claimed to be useful in the genome-inspired development of new probiotic formulations.

In this study, *Bifidobacterium* and *Enterococcus* isolates were recovered from the faeces of Cameroonian infants, and their whole-genome sequences were examined to unravel the genetic basis for their prospective beneficial abilities, antimicrobial activities and safety.

## Methods

### Study participants, faecal sample collection and isolation of bacteria

Mothers (without gastrointestinal disorders, illness in the previous 7 days or antibiotic treatment in the previous 14 days) and infants aged 0–5 months were recruited in the health district of Dschang (Menoua Division, West Region, Cameroon) following parental written informed consent. The infant cohort consisted of vaginally delivered full-term healthy babies, breast-fed and/or mixed-fed (but not receiving probiotic formula) (Table S1, available in the online version of this article).

Fresh infant faecal samples (from diapers immediately after defecation) were aseptically collected in labelled faeces collectors and kept in iceboxes. They were subsequently transported to the laboratory within 2 h and frozen at −80 °C in de Man, Rogosa and Sharpe (MRS, Lab M, UK) broth supplemented with glycerol (30% v/v) until shipping to the Technical University of Munich for processing (Intestinal Microbiome, ZIEL-Institute for Food and Health, TUM, Germany).

For isolation, 26 faecal glycerol stocks were tenfold serially diluted (1 g, up to 10^−5^), and the dilutions were plated (100 µl) onto MRS agar (BD Difco™, Becton, Dickinson and Company, USA) without any additives or supplemented with l-cysteine hydrochloride (0.05%, Sigma-Aldrich, UK) and mupirocin (0.005%, Sigma-Aldrich, UK). The plates were then incubated at 37 °C for 48 h, aerobically or in an anaerobic chamber (Whitley A95 Anaerobic Workstation, Don Whitley Scientific Limited, UK). For each sample, three colonies from each dilution were randomly selected, visually examined for colony and cell morphology characteristics and streaked to purity on the isolation medium. Pure cultures were stored at −80 °C in cryogenic tubes containing MRS with 30% glycerol.

### Genomic DNA extraction, whole-genome sequencing and data processing

Total DNA was extracted from bacterial cells (pellet of 48-h-old culture) using the FastDNA™ SPIN kit (MP Biomedicals, USA) after lysis in the FastPrep-24 instrument (MP Biomedicals, USA) at a speed of 6.0 m s^–1^ for 40 s. Partial 16S rRNA gene sequencing was performed using primers 5′-AGA GTT TGA TCC TGG CTC AG-3′, 5′-AGA GTT TGA TCA TGG CTC AG-3′ and 5′-ACG GTT ACC TTG TTA CGA CTT-3′ at Eurofins Genomics, Ebersberg, Germany. Preliminary identification of the isolates was performed with sina aligner (v.1.2.12) [29].

Based on the results of the preliminary identification, we selected *Bifidobacterium* and *Enterococcus* isolates and subjected them to whole-genome sequencing on either Illumina MiSeq (samples labelled ‘PM’) or Illumina NextSeq2000 platform (samples labelled ‘LH-CAM’) (read length 2×150 bp, average sequencing coverage of 216×) at the Quadram Institute Bioscience (Norwich, UK). Sequencing reads were pre-processed with fastp v0.20 (samples ‘PM’) and fastp v0.23 (samples ‘LH-CAM’) [30]. Draft genome assemblies were generated using SPAdes v.3.14.1 with ‘--careful’ option (‘PM’ samples) [31] and Unicycler v.0.4.9 with the ‘--mode conservative’ option (‘LH-CAM’ samples) [32]. Contigs below 1000 bp were filtered out of all assemblies. We estimated genome completeness and contamination using CheckM v.1.2.0 [33] and retained sequences with completeness >98% and contamination <1%. GTDB-Tk v.2.3.2 [34] was used to classify all genomic sequences to the strain level, and python3 module pyANI v.0.2.10 with default settings was used to calculate the average nucleotide identity (ANI) values [35]. Species delineation cut-off was set at 95% identity [36]. Sequences showing identity values above 99.9% from the same sample were considered identical and were removed from further analysis [37]. After such processing, the final dataset comprised 36 unduplicated *Bifidobacterium* and *Enterococcus* genomes (Table S2). All assemblies were annotated using Prokka v.1.14.6 [38]. In addition, previously assembled publicly available sequences of selected type strains *Bifidobacterium* and *Enterococcus* were retrieved from the NCBI Genome database.

### Computational analysis

The multiple sequence alignment generated with GTDB-Tk was used to assess the phylogenetic relationship of strains included in the analysis. Model testing and subsequent maximum likelihood estimation (best-fit model LG+F+R3) were performed in IQ-TREE v.2.0.5 [39]. For selected *Bifidobacterium* and *Enterococcus* isolates from different infants showing ANI values above 99.9%, snippy v.4.6.0 [40] was used to map the raw sequencing reads against reference assemblies, namely *Bifidobacterium animalis* subsp. *lactis* ATCC 22563^T^ and *Enterococcus faecalis* PM12. Carbohydrate metabolism properties of strains included in the dataset were profiled using a standalone version of dbCAN3 [41] with ‘--hmm_cov 0.50’ option. Prediction of the presence of HMO clusters in *Bifidobacterium* was performed by comparing genomes included in the final dataset to known bifidobacterial protein sequences using local blastp (e-value <1e^−50^). HMO clusters were annotated ‘present’ if all cluster components were identified at the above homology level. Incomplete clusters (more than three locally clustered genes) were annotated as ‘partially present’.

The presence of putative bacteriocin and secondary metabolite biosynthesis gene clusters in the assemblies was predicted using antiSMASH v.7.0 [42] and BAGEL4 [43]. MobileElementFinder v.1.0.3 [44] and VirulenceFinder 2.0 database tool [45] (with a threshold of at least 99% similarity) were used to assess the presence of plasmids, insertion sequences (IS) and virulence factors in our dataset.

The Comprehensive Antibiotic Resistance Database (CARD, v.3.2.9) (perfect or strict hits with at least 95% amino acid homology considered significant) [46,47] and the ResFinder v.4.4.2 tool incorporated in the MobileElementFinder package [48] (at least 99% identity threshold) were used for the *in silico* identification of antibiotic resistance genes in the analysed genomes. The standalone version of PHAge Search Tool with Enhanced Sequence Translation [49] implemented in Docker Compose v.2.30.3 [50] was employed as a predictive tool for the identification of prophages within bacterial genomes. The completeness level of all predicted prophage regions was evaluated using CheckV v.1.0.3 [51] with an ‘end-to-end’ parameter and the database v.1.5. To identify potential ARG and virulence factors in prophage sequences, we compared them to the CARD and VirulenceFinder databases, as above.

The annotated files produced by Prokka were used to manually search for the presence of genes and gene clusters involved in tolerance to acid and bile salts, general stress resistance, adhesion and host GIT adaptations.

R v.4.2.3 [52] with packages factoextra v.1.0.7 [53] and ComplexHeatmap [54], as well as iTOL v.6.8.1 [55], were used for data visualization.

## Results

### General genomic features and relatedness of isolates recovered from Cameroonian infants

The purpose of this study was to define the beneficial potential of bifidobacteria and enterococci isolated from Cameroonian infants using an *in silico* approach. A total of 150 isolates were obtained from the faecal samples. After preliminary identification based on partial 16S rRNA gene sequencing, we performed whole-genome sequencing on *Enterococcus* and *Bifidobacterium* isolates, which constituted the majority of the recovered isolate pool (55 and 30%, respectively). Following processing and dereplication, we included 36 genomic sequences in the final dataset. Genome assembly produced sets of contigs ranging from 11 to 138 per strain (Table S2). The G+C content ranged from 37.0 to 38.2% for *Enterococcus* strains and 56.2 to 62.3% for *Bifidobacterium* strains. Consistent with previous reports [56], genome sizes ranged from 1.89 to 2.46 Mb for bifidobacteria (average of 2.22 Mb) and 2.68 to 3.21 Mb for enterococci (average of 2.91 Mb). Differences in genome sizes were reflected in the number of predicted CDS, which was lower in bifidobacteria compared to enterococci, with an average of 1858 and 2751, respectively.

**Fig. 1.**
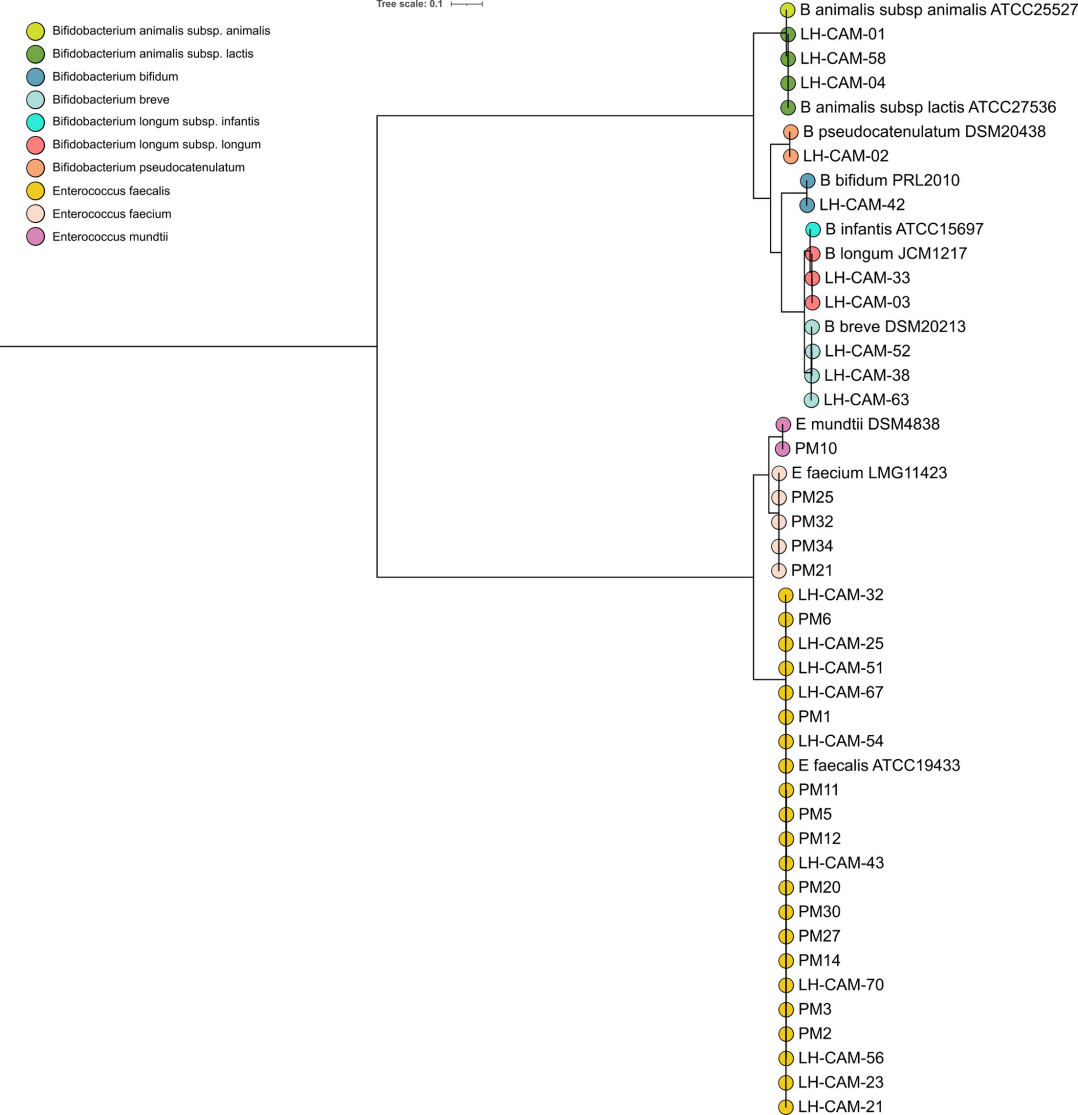
Relatedness of isolates recovered in this study to bacterial type strains representative of infant-associated species of *Bifidobacterium* and *Enterococcus*. The phylogeny was constructed from the GTDB-tk marker gene alignment using the maximum likelihood estimation based on the best-fit model LG+F+R3. The tree was rooted mid-point. The node colours denote cluster membership of analysed isolates.

Together with ten genomic sequences representative of type strains *Bifidobacterium* and *Enterococcus* species, genomes constituting our final dataset were subjected to relatedness and gene content assessment to gain insights into their global genomic traits. The phylogeny (Fig. 1) and the ANI analysis (Table S3) indicated clustering into eight main groups: *B. animalis*, with strains belonging to subsp. *lactis* identified in our dataset, *Bifidobacterium longum* subsp. *longum*, *Bifidobacterium breve*,*Bifidobacterium bifidum*, *Bifidobacterium pseudocatenulatum*, *E. faecalis*, *Enterococcus faecium* and *Enterococcus mundtii*. Based on the ANI values (ANI >99.9%) and snippy analysis of selected genomes, we identified what we considered to be clonal strains belonging to *B. animalis* subsp. *lactis* (<26 SNPs) and *E. faecalis* (1 SNP) taxa in samples from different infants (Table S4).

### Functional annotation of bifidobacterial and enterococcal genomes – potential for carbohydrate utilization

**Fig. 2.**
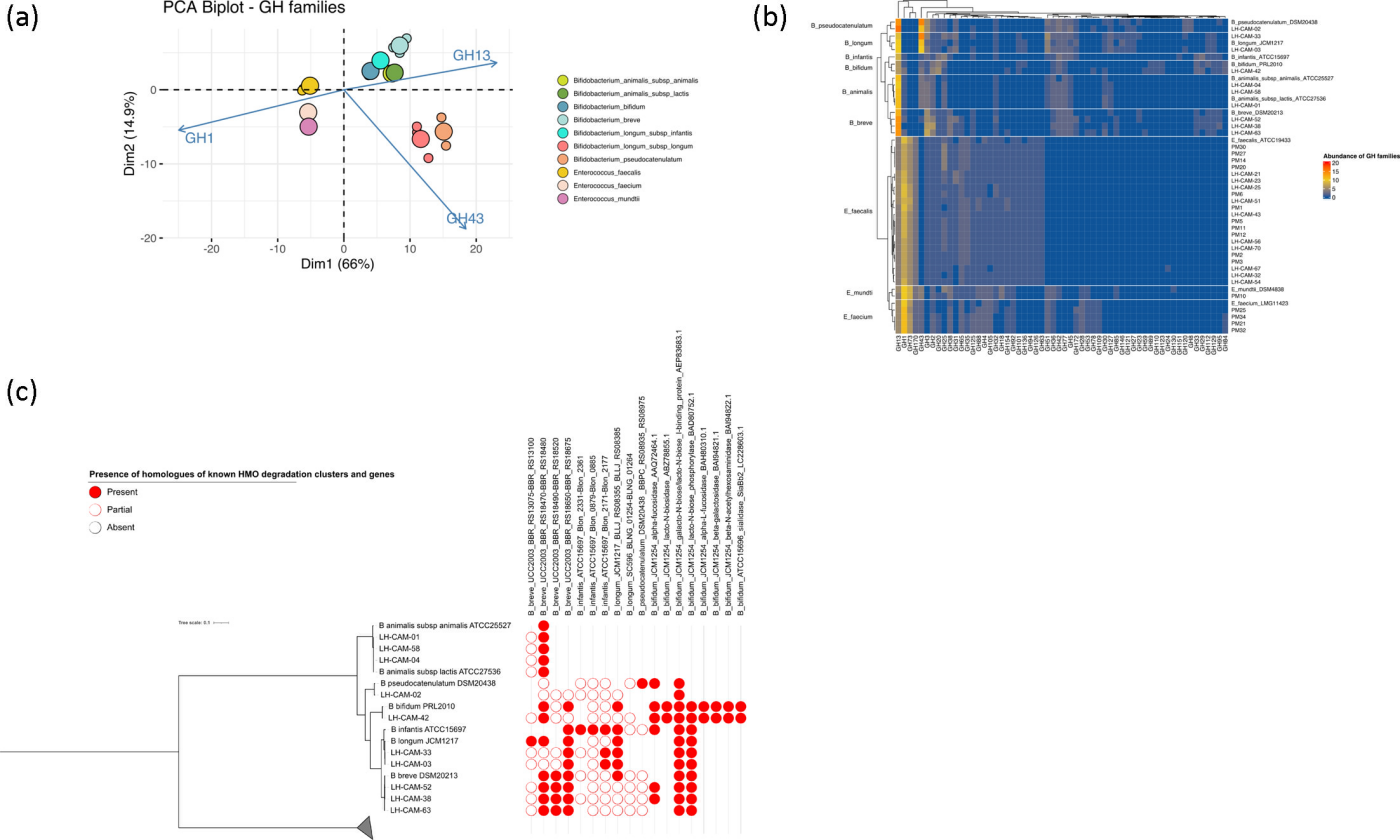
Genomic prediction of carbohydrate metabolism properties of strains analysed in this study. (**a**) Principal component analysis based on the predicted GH family abundance data grouped by bacterial species. The top three contributing variables are marked on the biplot. (**b**) Heatmap showing the abundance of predicted GH families in the strains included in the dataset. (**c**) Diagram depicting the presence and absence of homologues of known HMO degradation clusters and genes in *Bifidobacterium* isolates used in this study (blastp, e-value <1e^−50^). HMO clusters were annotated ‘present’ if all cluster components were identified at the above homology level. Incomplete clusters (more than three locally clustered genes) were annotated as ‘partially present’.

Different infant-associated bacterial taxa have previously been shown to possess particular carbohydrate metabolism capabilities, which are important for dietary substrate degradation and the development of cooperative microbial communities [57,60]. We therefore investigated carbohydrate-active enzyme family repertoires, and specifically the presence of glycoside hydrolases (GH), in *Bifidobacterium* and *Enterococcus* genomes, as well as the genomic potential of bifidobacteria to metabolize HMOs (Fig. 2, Tables S5 and S6).

On average, we identified 18 different GH families in *Bifidobacterium* sequences, with an average of 46 GH genes (2.1% of CDS) per genome, consistent with previous reports [26,61]. The predominant GH family identified in *Bifidobacterium* was GH13, comprising enzymes capable of hydrolysing α−1,4-glycosidic linkages in starches and similar substrates [62], while the second most abundant GH family detected in the genomes of *B. longum* and *B. pseudocatenulatum*, but not in other bifidobacterial species, was GH43 containing enzymes involved in the degradation of aryl glycosides [63]. Other notable GH families included GH3, GH2 and GH42, which encompass enzymes capable of hydrolysing a wide variety of plant cell wall glycans, with members of GH2 and GH42 families also implicated in the metabolism of lactose, a disaccharide abundant in human milk. We also identified putative GH33 exo-sialidases in the genomes of *B. bifidum* and *B. breve*, which indicates that these strains may be able to directly utilize host glycans and metabolize free sialic acid (Fig. 2a, b, Table S5) [64].

In enterococci, an average of 36 different GH families were identified, with an average of 47 genes per genome (1.3% of CDS). Genes identified as members of the GH1 family, which contains enzymes with β-glucosidase and β-galactosidase activity, were the most abundant. Consistent with previous findings, other highly represented GH families included GH73, which represents enzymes with suggested peptidoglycan hydrolase activity [65,66] and GH13 (Fig. 2a, b, Table S5).

Since the ability to metabolize HMOs is an important functional property of several early life-associated *Bifidobacterium* species, namely *B. breve*, *B. infantis*, *B. longum*, *B. pseudocatenulatum* and *B. bifidum* [67,71], we investigated the presence of known bifidobacterial HMO metabolism genes and gene clusters in our genomic dataset. Consistent with previous reports, the results of this analysis revealed strain- and species-specific differences in the distribution of putative homologues of clusters and genes implicated in HMO metabolism in our *Bifidobacterium* strains [26,72].

The metabolism of lacto-*N*-tetraose (LNT) and lacto-*N*-neotetraose (LNnT) has been linked to specific metabolic pathways in different bifidobacterial species and strains. The ability of type strain *B. infantis* ATCC 15697^T^ to degrade these HMOs has been shown to be regulated by a global transcription factor NagR acting as a regulator for multiple gene clusters, including *nag* (Blon_0879–0885), *lnp* (Blon_2717–2177) and H1 (Blon_2331–2361) [73]. We identified homologues to the *lnp* cluster in the two isolated Cameroonian *B. longum* strains (LH-CAM-03 and LH-CAM-33), with partial homology to the H1 cluster recorded only in *B. longum* LH-CAM-33. Additionally, *B. pseudocatenulatum* LH-CAM-02 and *B. breve* LH-CAM-38 showed partial homology to all three clusters (Fig. 2c, Table S6).

Similarly, the metabolism of LNT and LNnT in *B. breve* UCC2003 has been shown to involve several clusters, namely *lnt* (BBR_RS13075-BBR_RS13100), *lac* (BBR_RS18470-BBR_RS18480), *nah* (BBR_RS18490-BBR_RS18520),and *lnp/lgt* (BBR_RS18650-BBR_RS18675) [69]. All Cameroonian *B. breve* genomes contained homologues to *lac*, *nah* and *lnp/lgt* clusters; however, only partial homology to the *lnt* cluster was detected in our *B. breve* strains (Fig. 2c, Table S6).

Degradation of fucosylated HMOs by bifidobacteria has been linked to the presence of particular enzymatic machinery containing an α1-3/4-fucosidase (GH29) and/or α1–2-fucosidase (GH95). A specific gene cluster for 2′-fucosyllactose metabolism has been described in *B. longum* SC569 (BLNG_01254-BLNG_01264), with homologous genes identified in *B. infantis* ATCC 15697^T^ and *B. pseudocatenulatum* DSM 20438^T^ [70]. In our dataset, we identified putative GH29 and GH95 fucosidases in *B. breve* LH-CAM-38, *B. breve* LH-CAM-52 and *B. bifidum* LH-CAM-42, with all three strains showing partial homology to the *B. longum* SC569 cluster (Fig. 2b, c, Tables S5 and S6).

Previous studies in *B. bifidum* have shown that six extracellular enzymes, namely α1,2-fucosidase, α1-3/4-fucosidase, lacto-*N*-biosidase, β1,4-galactosidase, β1,3-*N*-acetylglucosaminidase and sialidase, as well as one intracellular phosphorylase and one transporter, play crucial roles in the metabolism of neutral HMOs in this species [74]. We detected the presence of homologues of all these genes in *B. bifidum* LH-CAM-42, with homology to the intracellular phosphorylase and the transporter alone identified in strains of other early-life-associated bifidobacteria (Fig. 2c, Table S6).

### Genomic antimicrobial, adhesion, stress-resistance and safety features of bifidobacteria and enterococci isolated from Cameroonian infants

A significant aspect of the initial probiotic strain selection is the assessment of antibiotic resistance patterns. In addition, a candidate strain must be able to tolerate acidic and bile-rich conditions of the GIT [75] and be able to adhere to the epithelium to successfully colonize the gut, thereby facilitating displacement of potentially pathogenic micro-organisms from host cells [76,77]. The production of antimicrobial compounds (e.g. bacteriocins) may enhance a strain’s ability to compete against other bacteria and potentially inhibit pathogenic microbes [78]. Therefore, we investigated the genomic potential of our bifidobacterial and enterococcal isolates to survive the passage through the GIT and their safety features.

#### Putative secondary metabolite production capabilities

We used antiSMASH and BAGEL4 to predict the presence of putative secondary metabolite biosynthesis gene clusters in our dataset (Table 1). In bifidobacteria, only one hit indicating putative production of class IV lanthipeptides was recorded in the genome of *B. pseudocatenulatum* LH-CAM-02 after antiSMASH analysis. Class IV lanthipeptides are young members of ribosomally synthesized and posttranslationally modified peptide (RiPP) subfamilies, described more recently and having no or only weak antibacterial activity [79]. In contrast, the presence of several different secondary metabolite biosynthesis gene clusters was predicted in enterococci. Overall, gene clusters for cytolysin ClyLI and CLyLs, enterolysin A, enterocin SE-K4, enterocin P, enterocin 96, propeptin, propeptin 2 and sactipeptides were found in *E. faecalis* genomes; enterocin A, enterolysin A, Bac 32, UViB, type III polyketide synthase (T3PKS) and sactipeptides in *E. faecium* genomes; and T3PKS, UViB, terpene, enterolysin A and sactipeptides in *E. mundtii* PM10 genome (Table 1).

#### Prediction of plasmids, mobile genetic elements, antibiotic resistance genes, virulence factors and prophage sequences

**Fig. 3.**
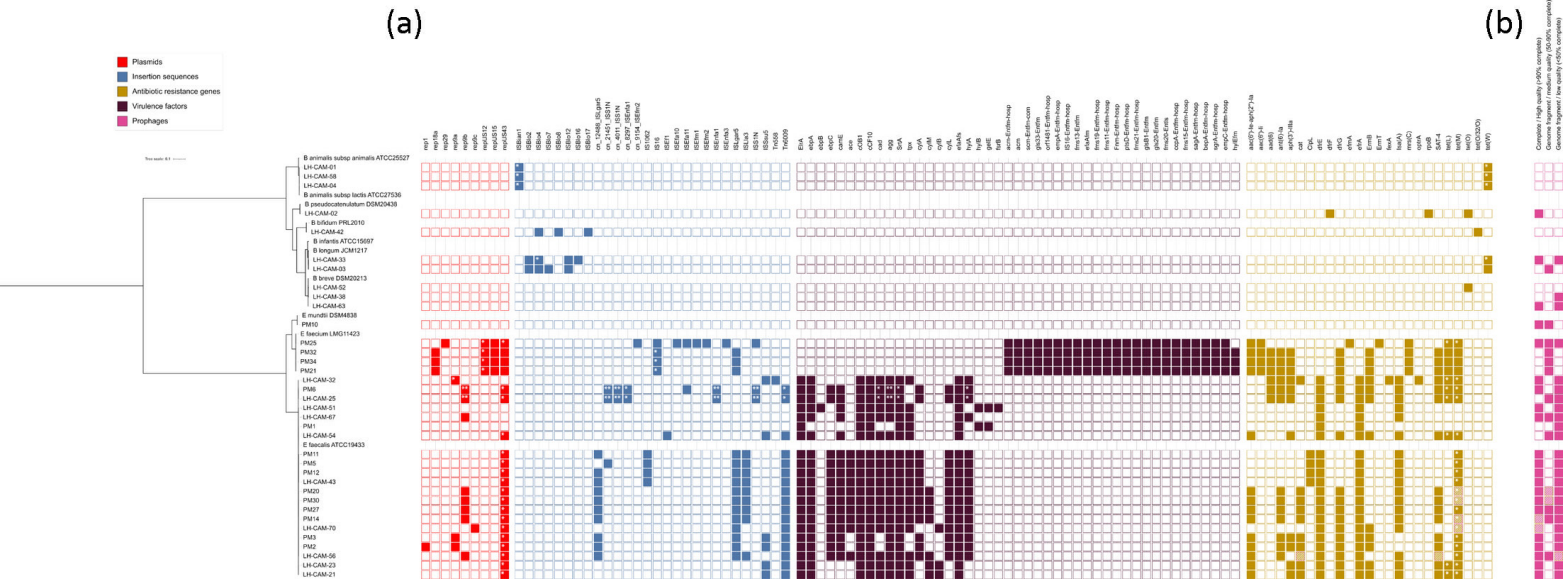
Genomic prediction of features potentially associated with virulence and antimicrobial resistance in the strains recovered in this study. (**a**) The coloured squares denote the presence of DNA regions encoding putative plasmids (red; MobileElementFinder, 99% identity), insertion sequences (blue; MobileElementFinder, 99% identity), virulence factors (maroon; VirulenceFinder, 99% identity) and antibiotic resistance genes (mustard; combined results from CARD and ResFinder databases, 95 and 99% amino acid identity, respectively). Asterisks denote features located on the same contig. (**b**) The coloured squares mark the presence of identified prophage sequences at various genomic integrity levels (pink; CheckV quality score as follows: high quality, >90% complete; medium quality, 50–90% complete; low quality, <50% complete). Striped squares in both the antibiotic resistance panel and the prophage panel denote corresponding putative prophage-mediated antibiotic resistance genes.

Assessment of genomic safety features is a crucial step in the characterization of potentially beneficial bacterial strains. Our results from such analysis indicate that bifidobacteria isolated from Cameroonian infants do not possess plasmids nor virulence factors but harbour putative tetracycline resistance genes [*tet(W*) and *tet(O*)] in their genomes, except for two *B. breve* strains, LH-CAM-38 and LH-CAM-68 (Fig. 3, Table S7). In addition, *B. lactis*, *B. longum* and *B. bifidum* strains were found to harbour IS homologous to previously reported various strains of *B. animalis* subsp. *animalis* and *lactis* [80] and *B. longum*, in particular *B. longum* NCC2704 [81].

Regarding enterococci, *E. mundtii* PM10 was interestingly found to be free of plasmids, IS, antibiotic resistance genes and virulence factors (Fig. 3, Table S7). Two *E. faecalis* strains (LH-CAM-51 and PM1) were found not to possess plasmids nor IS, but they harboured various ARG and virulence factors. The majority of *E. faecalis* genomes were found to contain homologues of genes encoding resistance to tetracyclines [*tet(M)*, *tet(L)*, *tet(S*)], lincosamides (*lsaA*), macrolides (*ermB*, *ermT*, *msrC*), aminoglycosides [*aac(6′)-Ii*, *aph(3′)-III*, *ant (6)-Ia*, *aac(6′)-aph(2″*)], streptogramins (*lsaA*, *msrC*), trimethoprim (*dfrG*) and chloramphenicol (*cat*, *optrA*). In addition, a plethora of virulence genes were detected, including biofilm formation-associated pili genes (*ebpA*, *ebpB*, *ebpC*, *SrtA*), adhesins (*ace*, *efaAfs*, *agg*, *acm*, *efaAfm*), sex pheromones (*cad*, *camE*, *cCF10*, *cOB1*), cytolysins (*cylB*, *cylL*, *cylM*), hyaluronidase (*hylA*, *hylB*), gelatinase (*gelE*), oxidative stress resistance (*tpx*), anti-phagocytic activity (*ElrA*) and quorum-sensing (*fsrB*). An even broader spectrum of virulence genes was detected in *E. faecium* genomes (Fig. 3, Table S7). All *Enterococcus* genomes were found to contain prophage sequences, with *E. faecium* strains harbouring 1.74 and *E. faecalis* strains harbouring 3.38 prophage fragments, on average (Fig. 3, Tables S9 and S10). We did not identify any virulence factors associated with prophage sequences, and it is worth noting, however, that we detected prophage-mediated ARGs in five out of 21 *E. faecalis* strains (23.8%), with tetracycline resistance gene *tet(M*) being the most commonly identified (Fig. 3, Table S11). Moreover, in the remaining *E. faecalis* and *E. faecium* strains, *tet(M*) and *tet(L*) genes were often located on plasmids.

#### Prediction of probiotic and stress-related genes in strains selected as safe

Based on the results of our *in silico* functional and safety screens, we selected 11 Cameroonian strains of interest, namely all *Bifidobacterium* strains and the *E. mundtii* PM10, and assessed their genomic potential for gut survival and stress response. This analysis revealed the putative presence of a vast array of genes important for cell survival and intestinal adhesion in all analysed genomes (Table S8). Among the acid tolerance genes identified, ATP-dependent protease proteolytic subunits (ATPase subunits a, b, c, alpha, beta, gamma and delta), Na^+^/H^+^ antiporter, l-lactate dehydrogenase, aspartate-semialdehyde dehydrogenase, glyceraldehyde-3-phosphate dehydrogenase, glucose-6-phosphate isomerase, pyruvate kinase and protein RecA were present in both *Bifidobacterium* and *Enterococcus*, with species-specific differences recorded for the components of phosphotransferase systems, i.e. cellobiose-specific enzymes, and ATP-dependent Clp protease ATP-binding subunits, among others. Not all bifidobacterial strains harboured the full spectrum of acid tolerance genes.

In terms of putative tolerance to bile salts, bile salt hydrolase, phosphoglycerate kinase, cytidine triphosphate synthase, chaperone protein DnaK and DnaJ, ornithine carbamoyltransferase and arginine repressor ArgR genes were found in all *Bifidobacterium* and the *Enterococcus*, while the arginine/ornithine antiporter ArcD was found exclusively in the *E. mundtii* PM10 genome. Among the genes responsible for adhesion, manganese ABC transporter substrate-binding lipoprotein, sortase D and tyrosine-protein kinases YveL and YwqD were only identified in *E. mundtii* PM10. Moreover, among the 12 adaptive stress response genes screened for universal stress protein, heat shock protein and cold-shock protein-encoding genes, eight were present in *E. mundtii* PM10, while only six of them were distributed among the *Bifidobacterium* species. Stress response protein YhaX, general stress proteins 13 and A and cold-shock protein 2, CspLA and CspD genes were present in *E. mundtii* PM10 only, while genes for groES and groEL chaperonines were found exclusively in *Bifidobacterium* strains. Interestingly, *E. mundtii* PM10 harbours more survival, adhesion and stress resistance genes than bifidobacteria.

## Discussion

The establishment of a healthy gut microbiota in early life plays an important role in maintaining immediate and long-term health. Alteration in microbiota composition in infancy, especially in LMIC communities, has been associated with several disease pathologies, such as diarrhoea, inflammatory bowel disease and necrotizing enterocolitis, thus increasing the risk of serious morbidity [11,12]. Thus, interventions based on the use of probiotics, prebiotics or symbiotics provide promising therapeutic options in LMICs [15,16]. However, it is important to consider screening geographically and population-relevant beneficial early-life gut microbiota members for next-stage development. In Cameroon, for instance, the prevalence of probiotic/synbiotic consumption is low, and even nil in some rural areas, with mainly dairy products containing probiotic strains or nutraceuticals, as well as probiotic infant formulas. Coupled with this, microbiome research has been largely limited to metagenomic studies investigating the effect of lifestyle and HIV infection on the gut microbiota composition [82,87]. Based on the scarcity of information on the health-promoting potential of regular members of the gut microbiota of Cameroonian infants, it is important to explore this ecological niche. We set out to fill this gap by isolating bifidobacteria and enterococci, sequencing their genomes and using *in silico* approaches with the aim of selecting local strains that may be used as probiotics in microbiota interventions in the future.

Upon isolation and whole-genome sequencing, we identified known infant-associated bifidobacteria and enterococci in our sample set. In terms of species diversity, our findings were consistent with previously published data from rural and semiurban areas of African LMICs, including Kenya [88], Ivory Coast, Malawi [89,90] and Zimbabwe [91]. Surprisingly, we detected both *Bifidobacterium* and *Enterococcus* strains characterized by a low number of SNP differences (<30) in samples from different infants. The *B. animalis* subsp. *lactis* ATCC 27536^T^ is a well-known probiotic used worldwide, and its presence in infants from our cohort can perhaps be explained by the consumption of probiotic products or nutraceuticals containing this strain by the mothers, while the presence of clonal *Enterococcus* strains in samples from different infants may be the consequence of an existing reservoir in the studied community.

Species and strains of *Bifidobacterium* are known to utilize HMOs, and this property likely contributes to their ability to function as a foundation genus within the wider context of the early-life microbiota [26,67, 69, 70, 92, 93]. HMOs represent a key nutritional component of breast milk, and substantial variations in HMO concentrations and profiles have been identified in women around the world [94,97]. Previous studies reported high proportions of milk containing a low abundance of α1–2-fucosylated structures for West and South Africa (37% in South Africa, 36% in Gambia and 32% in Ghana) compared with values for other African countries, including Namibia (17%) and Malawi (25%) [96,97]. To the best of our knowledge, no data exist on the oligosaccharide concentrations and profiles in breast milk from Cameroon; our analysis, however, identified *Bifidobacterium* strains, and *B. breve* and *B. bifidum* in particular, with genomic potential to metabolize a variety of HMOs, including fucosylated structures. Given that the presence of genes in the bacterial genomic sequence does not always result in a growth phenotype on the specified HMOs, experimental selection of robust HMO metabolizers as candidates for probiotic development will be a crucial next step.

Potentially, probiotic strains intended for commercial use should fulfil safety criteria, as per the European Food Safety Authority guidelines. Their genomes should be free from antibiotic-resistance genes or genes coding for known virulence factors such as toxins, invasion and adhesion factors [98]. AMR determinants located in the proximity of transposable elements or falling inside bacterial plasmids could contribute to the spread of AMR [99,100]. In our study, the *in silico* analysis showed that *B. breve* LH-CAM-38, *B. breve* LH-CAM-63 and *E. mundtii* PM10 did not harbour plasmids, mobile genetic elements, ARG or virulence factors but contained prophage sequences. Several *Bifidobacterium* strains were found to possess resistance genes either alone or together with a number of IS, which is in line with previous findings [101,103]. While the presence of IS flanking ARG has been implicated in the promotion of AR gene transfer between micro-organisms [104,105], previous studies have shown that the probability of gene transfer in *Bifidobacterium* possessing IS is extremely low [106,107]. The analysed *E. faecalis* and *E. faecium* strains were all found to harbour plasmids, IS, ARG, virulence factors and prophage sequences, consistent with previous literature reports showing that members of the *Enterococcus* and *Lactobacillus* genera carry AR and virulence genes either on plasmids, within prophage fragments or in the proximity of conjugative transposons [108,110].

The production of antimicrobial compounds such as bacteriocins contributes to higher niche competitiveness and inhibition of intestinal pathogens, a property regarded as a probiotic trait. Especially during the early-life developmental window, the production of bacteriocins by beneficial microbiota members can facilitate efficient colonization and ecosystem structuring. The screen for the secondary metabolite biosynthesis clusters among bifidobacteria predicted the presence of class IV lanthipeptide biosynthetic cluster only in one isolate, *B. pseudocatenulatum* LH-CAM-02. This result is consistent with the findings of Yu *et al*. [111] and the general consensus that bifidobacteria are overall poor sources of bacteriocins [112,115]. In contrast, enterococcal genomes encoded several diverse bacteriocin gene clusters, ranging from class I to III bacteriocins. This is in agreement with previous findings reporting that bacteriocin-encoding genes are widely disseminated among enterococci of different origins [116,118]. Notably, the *E. mundtii* PM10 strain, free from AR genes and virulence factors, showed genomic potential to produce T3PKS and terpene alongside the bacteriocins UViB and enterolysin A. Polyketide synthases have potential applications as anti-infective, anti-tumour and immunosuppressive agents. They have also been associated with bacterial viability and antibacterial activity of the producing bacteria in the intestinal environment [119,121]. Terpenes have been shown to play a significant role in treating various types of diseases through their anticancer, antimicrobial, anti-inflammatory, antioxidant, antiallergic, neuroprotective, anti-aggregator, anti-coagulation, sedative and analgesic effects [122,123].

To be able to exert their health-promoting effects on the host, bacteria should survive gastrointestinal transit and transiently colonize the GIT, which is also a prerequisite for bacteria termed probiotics. Indeed, the harsh conditions of the stomach and small intestine, especially exposure to oxygen or other oxygen-derived free radicals, organic acids and bile, as well as osmotic stress, can have a negative impact on cell viability and hence on probiotic functionality [124]. Consistent with previous reports [28,125], we detected a plethora of genes related to acid and bile salt tolerance, general response to stress and oxidative stress and temperature in our *Bifidobacterium* strains and *E. mundtii* PM10, indicating their high potential for survival in the GIT. These strains ought to be further characterized for their phenotypic probiotic and immune-modulatory properties, complemented with clinical trials.

## Conclusion

In this study, we isolated and genomically characterized a selection of bifidobacteria and enterococci from the intestinal microbiota of healthy Cameroonian infants. Screening genomes for beneficial as well as safety properties revealed that strains belonging to *B. bifidum*, *B. longum* subsp. *longum*, *B. breve*, *B. pseudocatenulatum*, *B. animalis* subsp. *lactis* and *E. mundtii* have genomic potential to be considered for further studies aiming at the development of customized probiotics from Cameroon in particular and LMICs in general. This is important for the creation of locally sourced microbiota-based therapies to improve infants’ health in specific regions. As a next step, it is important to define the applicability of these promising strains as therapeutics through phenotypic characterization and clinical trials. Moreover, the results of this study will significantly complement the information available on the intestinal microbiota composition of infant cohorts from underrepresented geographical areas of the world.

## Abbreviations

AMR: antimicrobial resistance
ANI: average nucleotide identity
ARG: AMR genes
CARD: Comprehensive Antibiotic Resistance Database
GH: glycoside hydrolase
GIT: gastrointestinal tract
HMOs: human milk oligosaccharides
IS: insertion sequences
LAB: lactic acid bacteria
LMICs: low- and middle-income countries
LNnT: lacto-*N*-neotetraose
LNT: lacto-*N*-tetraose
MRS: de Man, Rogosa and Sharpe
NCBI: National Center for Biotechnology Information
RiPP: ribosomally synthesized and posttranslationally modified peptide
SCFAs: short-chain fatty acids
T3PKS: type III polyketide synthase
